# Manipulation of acoustic wavefront by gradient metasurface based on Helmholtz Resonators

**DOI:** 10.1038/s41598-017-10781-5

**Published:** 2017-09-06

**Authors:** Jun Lan, Yifeng Li, Yue Xu, Xiaozhou Liu

**Affiliations:** 1 College of Computer Science and Technology, Nanjing Tech University, Nanjing, 211800 P. R. China; 2 Key Laboratory of Modern Acoustics, Ministry of Education, Nanjing University, Nanjing, 210093 P. R. China; 3 Key Laboratory of Modern Acoustics, Ministry of Education, Institute of Acoustics and School of Physics, Nanjing University, Nanjing, 210093 P. R. China

## Abstract

We designed a gradient acoustic metasurface to manipulate acoustic wavefront freely. The broad bandwidth and high efficiency transmission are achieved by the acoustic metasurface which is constructed with a series of unit cells to provide desired discrete acoustic velocity distribution. Each unit cell is composed of a decorated metal plate with four periodically arrayed Helmholtz resonators (HRs) and a single slit. The design employs a gradient velocity to redirect refracted wave and the impedance matching between the metasurface and the background medium can be realized by adjusting the slit width of unit cell. The theoretical and numerical results show that some excellent wavefront manipulations are demonstrated by anomalous refraction, non-diffracting Bessel beam, sub-wavelength flat focusing, and effective tunable acoustic negative refraction. Our designed structure may offer potential applications for the imaging system, beam steering and acoustic lens.

## Introduction

Recent years have witnessed intense investigation of acoustic metasurface capable of realizing general wavefront modulation^1–9^. According to the generalized Snell’s law^10^, the angles of reflected, refracted and diffracted waves can be artificially operated by the metasurface with gradient change of acoustical phase^7–9, 11, 12^. In addition, the fascinating phenomena and capabilities, such as acoustic bending^13, 14^, anomalous refraction^15^, ultrathin flat lens^4, 16–18^, conversion of propagating wave to surface wave^19–21^, tunable acoustic negative refraction^22^, etc., have been exhibited by different kinds of gradient metasurfaces. The research on acoustic metasurface has significantly inspired the review of the fundamental physics and broadened the horizon for acoustic waves. In order to manipulate wavefront flexibly, some methods by employing the acoustic metasurface with transversal gradient refractive index or gradient velocity have been demonstrated. For the construction of the acoustic metasurface with gradient refractive index, previous studies have focused on space folding unit cells such as labyrinthine and interdigitated shapes^16, 17, 23–25^, which successfully exhibit the transversal relative refractive index in a gradient distribution and manipulate the acoustic wavefront through appropriately selecting the folding degree to delay the phase of acoustic wave. However, all of these metasurfaces have complex structures since the gradient refractive index is determined by the folding degree or the size of unit cell. In addition to adjusting the refractive index, tailoring the acoustic velocity is another effective method to realize the wavefront manipulation^12^. Recently, the metasurface on the basis of the two-dimensional (2D) pentamode metamaterial has been introduced and fabricated^12^. This kind of metasurface with gradient velocity provides a new design methodology for acoustic wave modulation and realizes a well matched impedance to improve the transmission efficiency. Successful wavefront manipulations, such as anomalous refraction, non-diffracting Bessel beam and flat lens have been demonstrated with the pentamode metasurface. However, this approach has high requirement for the metasurface structure. So far, broaden the application of the existing gradient metasurfaces is still a challenge. For the metasurface based on space folding unit cells, despite the acoustic phase can manipulated flexibly, the impedance matching between the metasurface and the background medium is relatively poor^25, 26^ and good matched impedance is realized only at the resonant frequency. For the pentamode metasurface, the impedance matching and broadband transmission are achieved, however, the high requirement of the structure restricts the flexibility of applications.

In this work, we design an acoustic metasurface capable of realizing general wavefront modulation based on the generalized Snell’s law for the acoustic refracted wave. The proposed metasurface comprises a series of unit cells based on Helmholtz resonators (HRs)^27–29^, which are composed of a decorated metal plate with four periodically arrayed HRs and a single slit at the right side. The designed metasurface exhibits the properties of broadband and high efficiency transmission compared with previous space folding metasurfaces^16, 17, 24, 25^. Besides, the discrete distribution of the acoustic velocity and the impedance matching can be easily realized by tailoring the slit width. Theoretical analysis and numerical calculation show that the proposed metasurface will be able to realize four distinct wavefront manipulations: anomalous refraction, non-diffracting Bessel beam, sub-wavelength flat focusing, and effective tunable acoustic negative refraction. All of these four properties are analyzed with the theoretical descriptions through the generalized Snell’s law. Our gradient velocity metasurface developed here helps to offer a new design methodology for acoustic wavefront engineering.

## Results

### Gradient acoustic velocity and impedance matching

**Figure 1:**
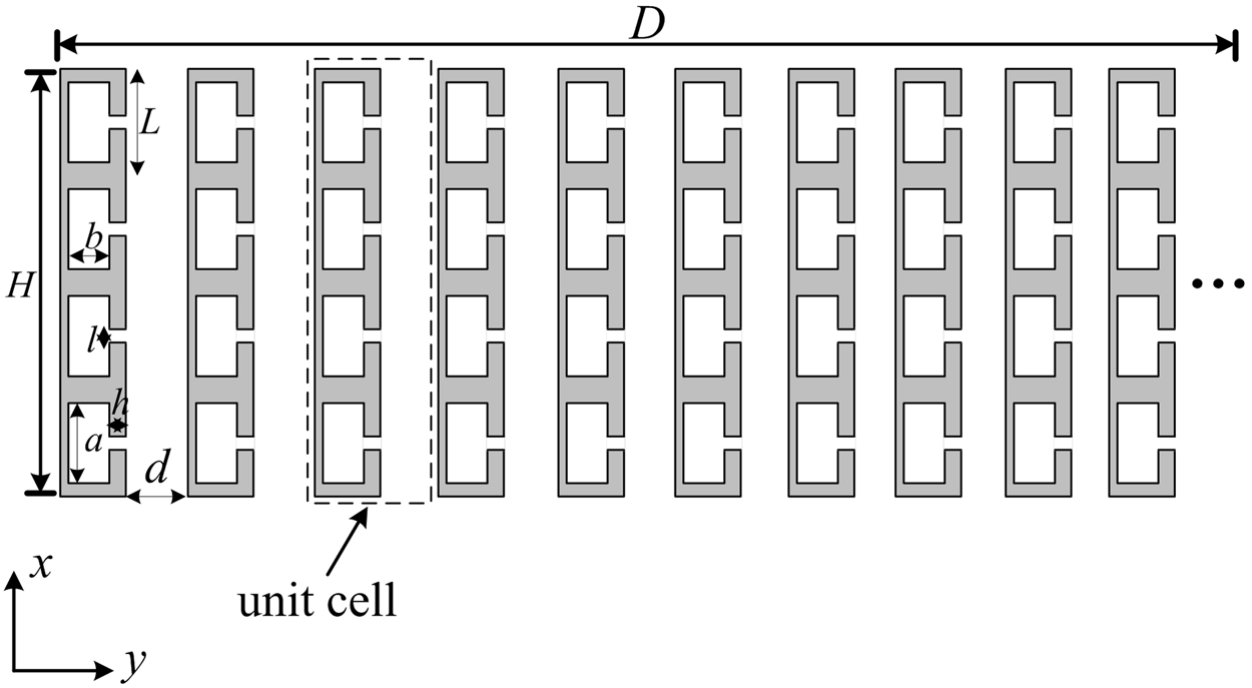
Schematic illustration of metasurface and its constituting elements. The black dotted box indicates the unit cell of the gradient metasurface.

We firstly illustrate the construction of the gradient acoustic velocity metasurface. Figure 1 is the schematic diagram of an acoustic metasurface in the *xy*-plane, in which the unit cells are located one by one along the *y*-axis. The proposed model introduces transversal gradient acoustic velocity instead of gradient refractive index to manipulate the refracted wave arbitrarily. The unit cell of the metasurface is shown by the black dotted box in Fig. 1, which is composed of a decorated metal plate with four periodically arranged HRs and a single slit at the right side. The height of the plate (*H*) and the periodic constant of HR (*L*) are 32 mm and 8 mm, respectively. The height (*a*) and width (*b*) of the cavity of HR are 6 mm and 2.5 mm, respectively. The length (*h*) and width (*l*) of the neck of HR are respectively 2 mm and 1 mm. Here, *d* and *D* are the widths of the right slit and the whole metasurface, respectively. An acoustic wave impinges normally on the metasurface along the +*x* direction. The background medium is air with density *ρ*_0_ of 1.21 kg/m^3^ and acoustic velocity *c*_0_ of 343 m/s.

In order to efficiently modify the radiation pattern of the transmitted sound wave, it is first of all necessary to illustrate how the velocity of the acoustic wave in metasurface can be engineered by HRs. The unit cell of the metasurface shown in Fig. 1 consists of an array of HRs, which exhibits an effective bulk modulus *B*_*eff*_ expressed as^30^

1 $$\frac{1}{{B}_{eff}}=\frac{1}{{B}_{0}}(1-\frac{F{{\omega }_{0}}^{2}}{{\omega }^{2}-{{\omega }_{0}}^{2}+i{\rm{\Gamma }}\omega }),$$

where *B*_0_ = *ρ*_0_ *c*_0_^2^ is the bulk modulus of the air, *F* = *ab*/*Ld* is the area ratio of the HR cavity to the slit section, *ω*_0_ and Γ are the resonant frequency and the intrinsic loss of the HR, respectively. Equation (1) implies that the unit cell of metasurface has two characteristic frequencies ${\omega }_{0}=1/\sqrt{{C}_{HR}{M}_{HR}}$ and ${\omega }_{n}={\omega }_{0}\sqrt{1+F}$, where *C*_*HR*_ = *ab*/*ρ*_0_ *c*_0_^2^ is the acoustic capacitance representing the spring action of the HR cavity, *M*_*HR*_ = *ρ*_0_ *h*_*eff*_/*l* is the acoustic mass corresponding to the mass of the air in the HR neck, and *h*_*eff*_ is the effective neck length^19, 31^. Here, the value of the resonant frequency of HR is *f*_0_ = *ω*_0_/2*π* = 7331 Hz. The real part of the effective bulk modulus is negative in the frequency range of *ω*_0_ < *ω* < *ω*_*n*_. According to the acoustic wave equation and Newton’s law, the existence of HRs does not affect the effective density^32^. Therefore, the effective acoustic velocity and wave vector of the unit cell of metasurface can be expressed as ${c}_{eff}=\sqrt{{B}_{eff}/{\rho }_{0}}$ and $k=\sqrt{{\omega }^{2}{\rho }_{0}/{B}_{eff}}$, respectively. For the frequency range of *ω*_0_ < *ω* < *ω*_*n*_, the wave vector is an imaginary number, and the acoustic wave attenuates rapidly in the +*x* direction and cannot propagate through the metasurface. For the other frequency ranges of *ω* < *ω*_0_ and *ω* > *ω*_*n*_, the wave vector is a real number, and the acoustic wave can propagate well in the metasurface. The reciprocal of the effective acoustic velocity in the unit cell of metasurface without intrinsic loss can be expressed as

2 $$\frac{1}{{c}_{eff}}=\sqrt{\frac{{\rho }_{0}}{{B}_{eff}}}=\sqrt{\frac{{\rho }_{0}}{{B}_{0}}(1+\frac{F{{\omega }_{0}}^{2}}{{{\omega }_{0}}^{2}-{\omega }^{2}})}.$$

Equation (2) indicates that: (i) As the frequency decreases from *ω*_0_ to 0, the effective velocity increases from 0 to the asymptotic value $\sqrt{{{c}_{0}}^{2}/(1+F)}$, which is smaller than the speed of sound in air *c*_0_. (ii) For the frequency range of *ω* > *ω*_*n*_, with the increasing of the frequency, the effective velocity decreases from infinity to *c*_0_. (iii) The acoustic velocity is affected by the area ratio *F* of the HR cavity to the slit section. For the frequencies below *ω*_0_, the value of effective velocity is inversely proportional to *F*. Through appropriately selecting the width of slit *d*, the value of *F* is changed simultaneously, and there is an inverse proportion relation between *d* and *F*. Therefore, in the frequency range of *ω* < *ω*_0_, the desired gradient acoustic velocity can be easily obtained by changing the width of slit *d*. The acoustic velocity distributions for the different wavefront manipulations will be demonstrated in detail later.

**Figure 2:**
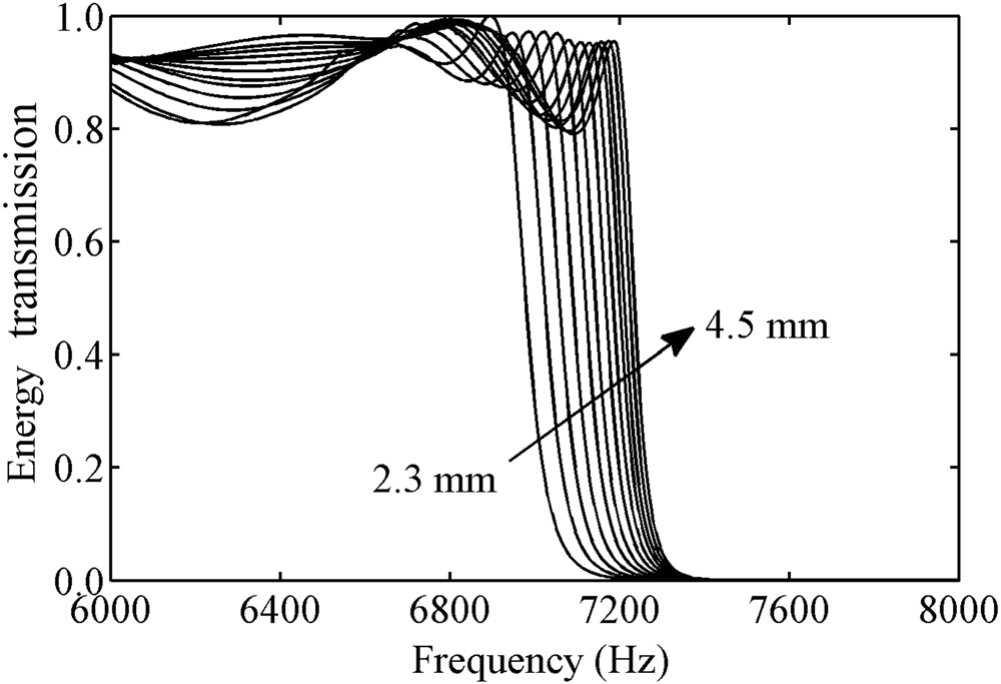
Energy transmission spectra for twelve metasurface units with different widths (from 2.3 to 4.5 mm).

For the purpose of designing an ideal acoustic metasurface with high efficiency transmission, it is crucial to keep the impedance matching between the metasurface and the background medium. Intuitively, the impedance of the proposed metasurface does not match with the background medium because of the sudden change of cross-sectional areas at the interface, i.e., *S*_*m*_ < *S*_*n*_, where *S*_*m*_ and *S*_*n*_ are the cross-sectional areas of the metasurface and the background medium at the inlet or outlet of a unit cell, respectively. In acoustics, the coefficients of reflection and transmission of an acoustic wave between two media are determined by the acoustic impedance (*Z*_*ac*_ = *ρc*/*s*), which is inversely proportional to the corresponding cross-sectional area^33, 34^. Therefore, in order to fulfill the impedance matching condition, either effective density or effective bulk modulus of the gradient metasurface needs to convert to a small value. Here, we manipulate the value of effective bulk modulus and make it smaller than that in the background medium, which would result in a small phase speed and slow wave. Equation (2) implies that, for the frequencies below *ω*_0_, the effective acoustic velocity in the metasurface is smaller than that in the background medium, which indicates a well impedance matching can be achieved at this frequency range. To verify this unique characteristic, the energy transmissions as a function of the frequency for the metasurface units with twelve different slit widths (from 2.3 to 4.5 mm) are illustrated in Fig. 2. The results show that the metasurface units could realize high efficiency transmission in the frequency range from 6000 to 7000 Hz, in which the transmission efficiency is nearly 0.9. Since the ratio of the cross-sectional areas between the metasurface and background medium is finite, the frequencies of transmission band are less than *f*_0_^33^. All of the above provide solid evidence that the acoustic impedance matching condition *ρ*_0_ *c*_*eff*_/*s*_*m*_ = *ρ*_0_ *c*_0_/*s*_*n*_ is satisfied. Therefore, this designed structure can be used as gradient acoustic metasurface with a relatively broad bandwidth and high efficiency transmission.

### Excellent wavefront manipulations of the metasurfaces

The gradient velocity metasurface introduces the phase discontinuity across the surface. Generally, the arbitrary wavefront manipulations can be guided and concluded by the generalized Snell’s law, and the transmitted wave across the acoustic metasurface can be expressed as follows^8^

3 $$\sin \,{\theta }_{t}(y)=\frac{1}{{k}_{0}}\frac{d{\rm{\Phi }}(y)}{dy}+\,\sin \,{\theta }_{i}(y),$$

where *θ*_*t*_(*y*) and *θ*_*i*_(*y*) are the angles of refraction and incidence, respectively. Φ(*y*) and *k*_0_ = *ω*/*c*_0_ are the phase factor and wave vector of air, respectively. It is found that the incident acoustic wave can be refracted arbitrarily by engineering the gradient of phase factor. When a plane acoustic wave (i.e.,*θ*_*i*_(*y*) = 0) propagates through the metasurface in the interesting frequency range, the gradient distributed and slowed acoustic velocity can be realized by suitably selecting the slit width of metasurface unit. The gradient phase can be expressed as $d{\rm{\Phi }}(y)/dy=H{k}_{0}{c}_{0}[d1/c(y)/dy]$, where *c*(*y*) is the velocity along the metasurface in *y* direction^12^. Substituting the gradient phase into Eq. (3), the refracted angle can be obtained as

4 $$\sin \,{\theta }_{t}(y)=H{c}_{0}\frac{d1/c(y)}{dy}.$$

Equation (4) indicates that the refracted angle is determined by the gradient of 1/*c*(*y*) directly. Therefore, by engineering the gradient velocity term, arbitrary wavefront modulations can be achieved, including anomalous refraction, non-diffracting Bessel beam, sub-wavelength flat focusing, and effective acoustic negative refraction.

### Anomalous refraction

**Figure 3:**
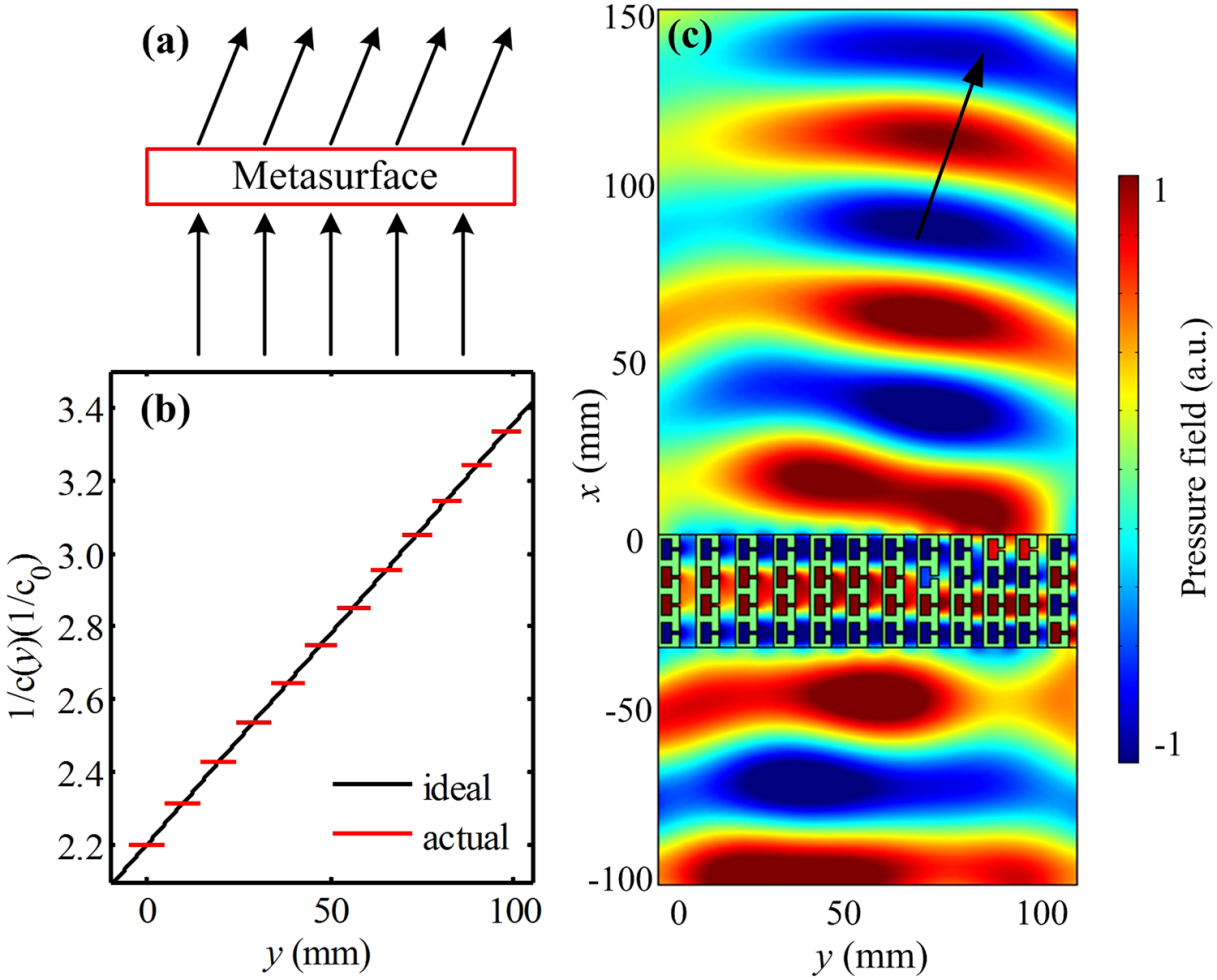
Acoustic metasurface for the anomalous refraction. (**a**) The concept schematic of the conversion process of generating anomalous refraction. (**b**) The reciprocal of the velocity distributions for the ideal metasurface (black solid line) and gradient metasurface (red solid line) at the working frequency range. (**c**) The simulated pressure field distribution of the gradient metasurface.

We will first demonstrate how to generate the anomalous refraction by the designed metasurface which is composed of twelve unit cells. Figure 3a illustrates the concept schematic of the conversion process of generating anomalous refraction. Considering the acoustic wave with normal incidence on the metasurface along the +*x* direction (i.e., *θ*_*i*_(*y*) = 0), the reciprocal of the velocity along *y* direction can be derived from Eq. (4)

5 $$\frac{1}{c(y)}=\frac{y}{H{c}_{0}}\,\sin \,{\theta }_{t}+\frac{1}{c(0)},$$

where the angle of the refracted wave is constant along the *y* direction and set to be *θ*_*t*_ = 20°. According to the mechanisms of gradient acoustic velocity and impedance matching as mentioned above, the acoustic velocity in the metasurface unit is depended on the slit width, and high efficiency transmission can be obtained when the acoustic impedance matching condition is satisfied. Thus, for the unit cells with the slit width of 4.5 mm and 2.3 mm, the corresponding ideal effective velocities are *c*_0_/2.2 and *c*_0_/3.4 at the working frequency range, respectively. In order to design an acoustic metasurface with the property of anomalous refraction, we choose the maximum velocity at the left edge and the minimum velocity at the right edge of the metasurface, which are *c*(0) = *c*_0_/2.2 and *c*(*D*) = *c*_0_/3.4, respectively, while the widths of slits are changed from 4.5 mm to 2.3 mm with a step of 0.2 mm. The reciprocal of the velocity distribution for the ideal metasurface is shown by the black solid line in Fig. 3b. Using our gradient metasurface, this ideal reciprocal of the velocity profile can be discretized into twelve stepwise zones, as shown by the red solid line in Fig. 3b. We depict the simulated acoustic pressure field under the normal incidence at the working frequency 6970 Hz in Fig. 3c. It should be observed that, when the plane wave propagates through the metasurface, the transmitted wavefront deflect from the incident direction. The propagation angle of the transmitted wave is coincident to that obtained with theoretical analysis, and the simulated result for the angle of the refracted wave corresponding to the black arrow is shown in Fig. 3c.

### Non-diffracting Bessel beam

**Figure 4:**
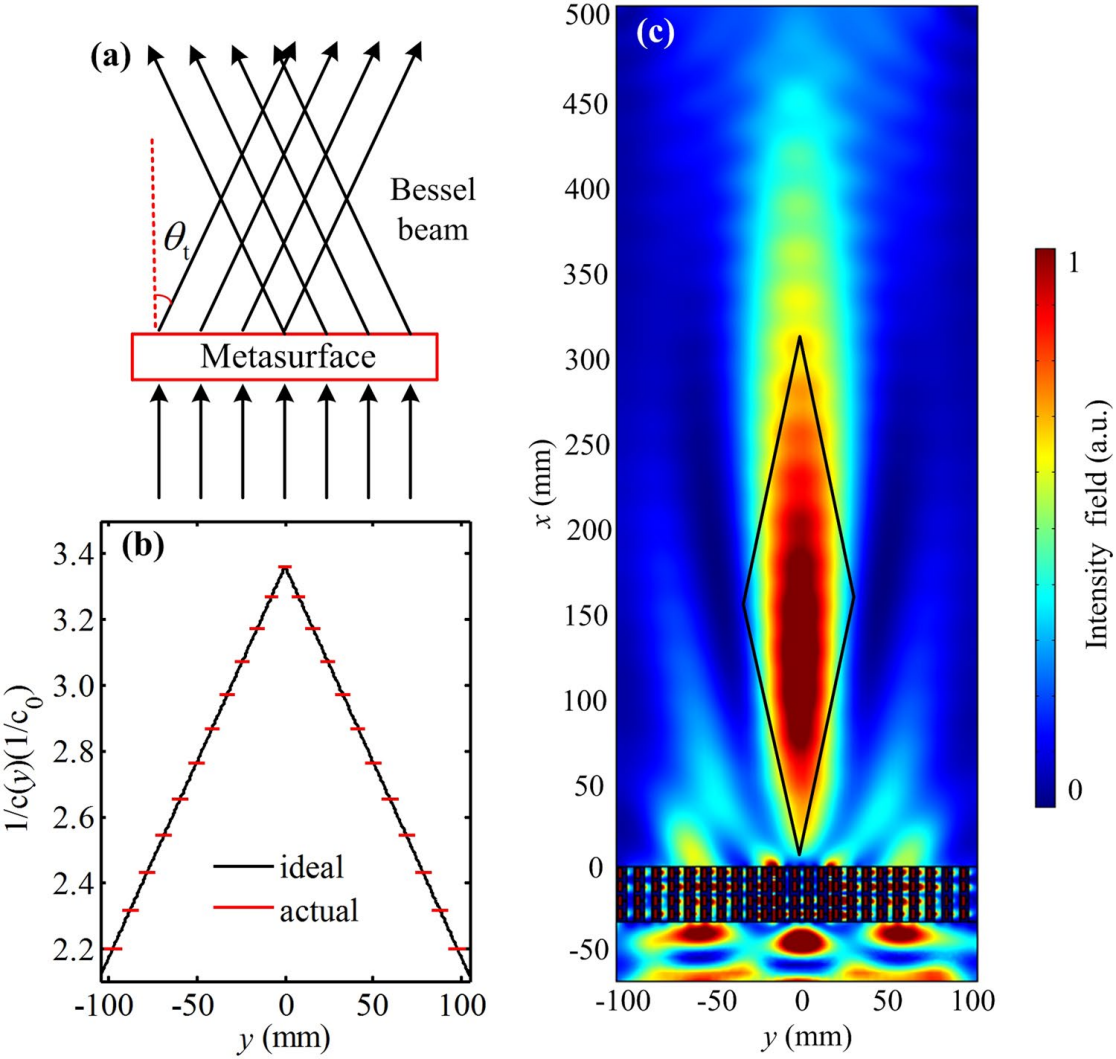
Acoustic metasurface for the non-diffracting Bessel beam. (**a**) The concept schematic of the conversion process of generating non-diffracting Bessel beam. (**b**) The reciprocal of the velocity distributions for the ideal metasurface (black solid line) and gradient metasurface (red solid line) at the working frequency range. (**c**) The simulated pressure intensity field distribution of the gradient metasurface.

It is well known that the non-diffracting Bessel beam can be realized by applying two plane beams with opposite propagating angles, and the overlapping region is the Bessel formation zone^35^. The corresponding concept schematic of the conversion process of generating non-diffracting Bessel beam is shown in Fig. 4a. The reciprocal of the velocity distribution for the ideal acoustic metasurface can be deduced as follows

6 $$\frac{1}{c(y)}=-\frac{|y|}{H{c}_{0}}\,\sin \,{\theta }_{t}+\frac{1}{c(0)},$$

where *θ*_*t*_ represents the base angle which is same as anomalous refraction and *c*(0) is the minimum velocity at the metasurface center. Figure 4b shows the reciprocal of the velocity distributions for the ideal metasurface (black solid line) and the gradient metasurface (red solid line) by setting *θ*_*t*_ = 20° and *c*(0) = *c*_0_/3.4, which indicates that the gradient velocity exhibits a perfect mirror symmetry. In this case, the designed metasurface is composed of 23 unit cells, and we choose the minimum velocity at the metasurface center and the maximum velocity at the metasurface edges to be *c*_*min*_(0) = *c*_0_/3.4 and *c*_*max*_(±*D*/2) = *c*_0_/2.2, respectively. Here, *D* is the width of the metasurface which is almost twice the size of anomalous refraction metasurface as mentioned above. The simulated acoustic pressure intensity field at the frequency 6970 Hz is shown in Fig. 4c. As expected, a non-diffracting Bessel beam propagating along the +*x* direction with a relatively long distance is observed. The simulated Bessel formation zone (black solid line) is in good qualitative agreement with the theoretical result (shown in Fig. 4a), which indicates that the effect of excellent non-diffracting Bessel beam can be achieved.

### Sub-wavelength flat focusing

**Figure 5:**
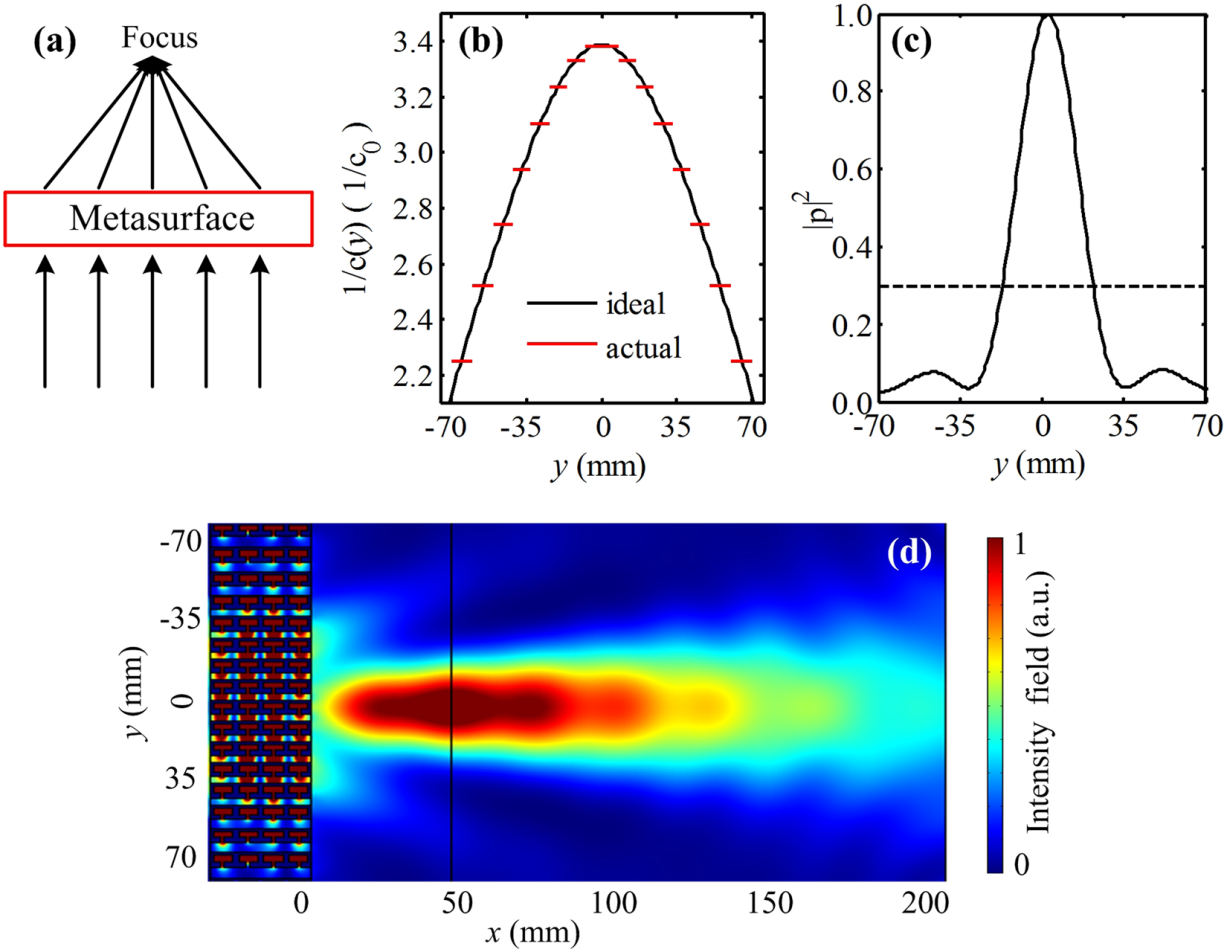
Sub-wavelength flat lens constructed by a gradient acoustic metasurface. (**a**) The concept schematic of the design of acoustic sub-wavelength flat lens. (**b**) The reciprocal of the velocity distributions for the ideal metasurface (black solid line) and gradient metasurface (red solid line) at the working frequency range. (**c**) Normalized transverse cross-sectional pressure intensity distributions along the *y*-axis at the focal point. (**d**) The simulated pressure intensity field distribution of the gradient metasurface.

We will now introduce how to design a sub-wavelength flat lens by the gradient metasurface. The designed metasurface can focus incident plane wave on a focal point (*x*_0_, 0), and the concept schematic is shown in Fig. 5a. The reciprocal of the velocity distribution along *y* direction can be expressed by the following equation

7 $$\frac{1}{c(y)}=-\frac{\sqrt{{y}^{2}+{{x}_{0}}^{2}}}{H{c}_{0}}+\frac{{x}_{0}}{H{c}_{0}}+\frac{1}{c(0)},$$

where *x*_0_ = 44 mm and *c*(0) = *c*_*min*_(0) = *c*_0_/3.4 are the abscissa of the focal point and the minimum velocity at the metasurface center, respectively. The maximum velocity at the metasurface edges is *c*_*max*_(±*D*/2) = *c*_0_/2.2. The desirable continuous reciprocal of the velocity for the ideal metasurface is plotted by the black solid line in Fig. 5b. Using our gradient metasurface with sixteen unit cells, the continuous reciprocal of the velocity can be discretized into sixteen stepwise zones at the working frequency range, as shown by the red solid line in Fig. 5b. The simulated pressure intensity field distribution of the gradient metasurface at the frequency 6970 Hz is shown in Fig. 5d, which indicates that the plane wave focusing is successfully realized. Moreover, it is found that the transmission energy is focused at (44, 0) with high amplitude, which is coincident to that obtained with theoretical analysis (*x*_0_, 0), demonstrating the excellent focusing effect. To quantify the performance of the sub-wavelength flat focusing, we further calculate the normalized transverse cross-sectional pressure intensity distribution along the *y*-axis at the focal point for the designed lens, the result of which is shown by the black solid line in Fig. 5c. The intensity at the focal point is about 3.4 times larger than the one of incident plane wave (black dash line), which provides clear confirmation that the excellent acoustic focusing effect can be obtained by the presence of designed metasurface.

**Figure 6:**
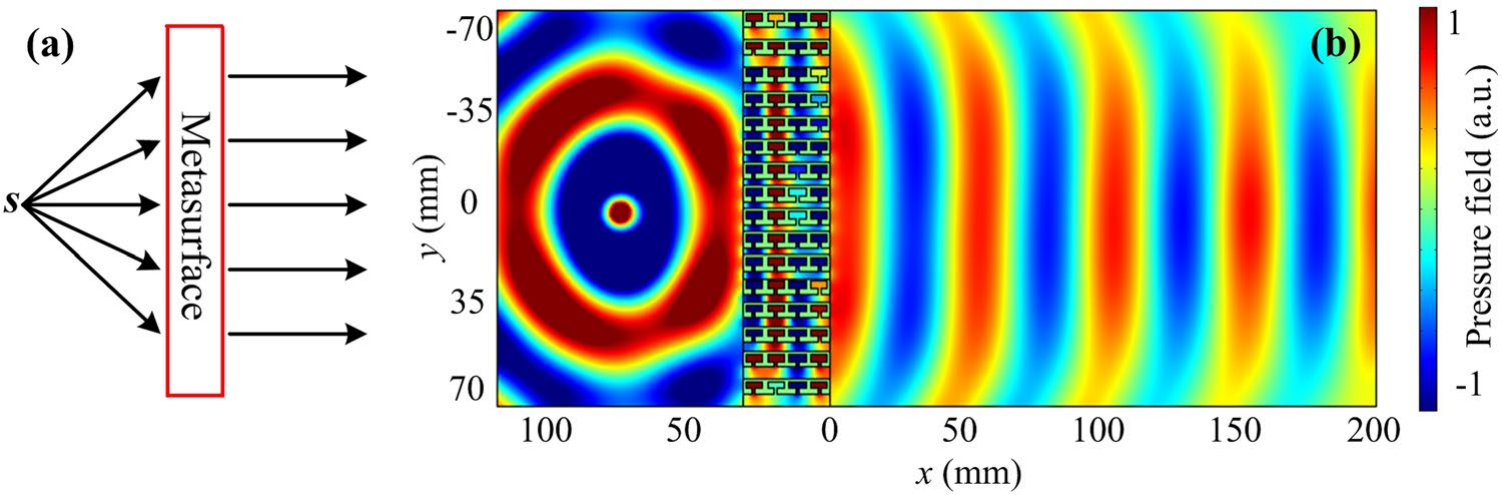
Sub-wavelength flat lens for cylindrical-to-plane-wave conversion. (**a**) The concept schematic of the cylindrical-to-plane-wave conversion. (**b**) Pressure field distribution of the gradient metasurface.

Next, according to the reciprocal theory, the designed sub-wavelength flat lens has the effect of efficient cylindrical-to-plane-wave conversion. The concept schematic of the conversion process is shown in Fig. 6a, a cylindrical wave is excited by a point source located at the left focal point of the metasurface. When the cylindrical wave propagates through the flat lens, a plane wave beam clearly emerges at the output space of the metasurface. Figure 6b shows the simulated pressure field distribution at the frequency 6970 Hz, which indicates that the cylindrical wave is converted into the plane wave efficiently by the gradient metasurface.

### Effective acoustic negative refraction

**Figure 7:**
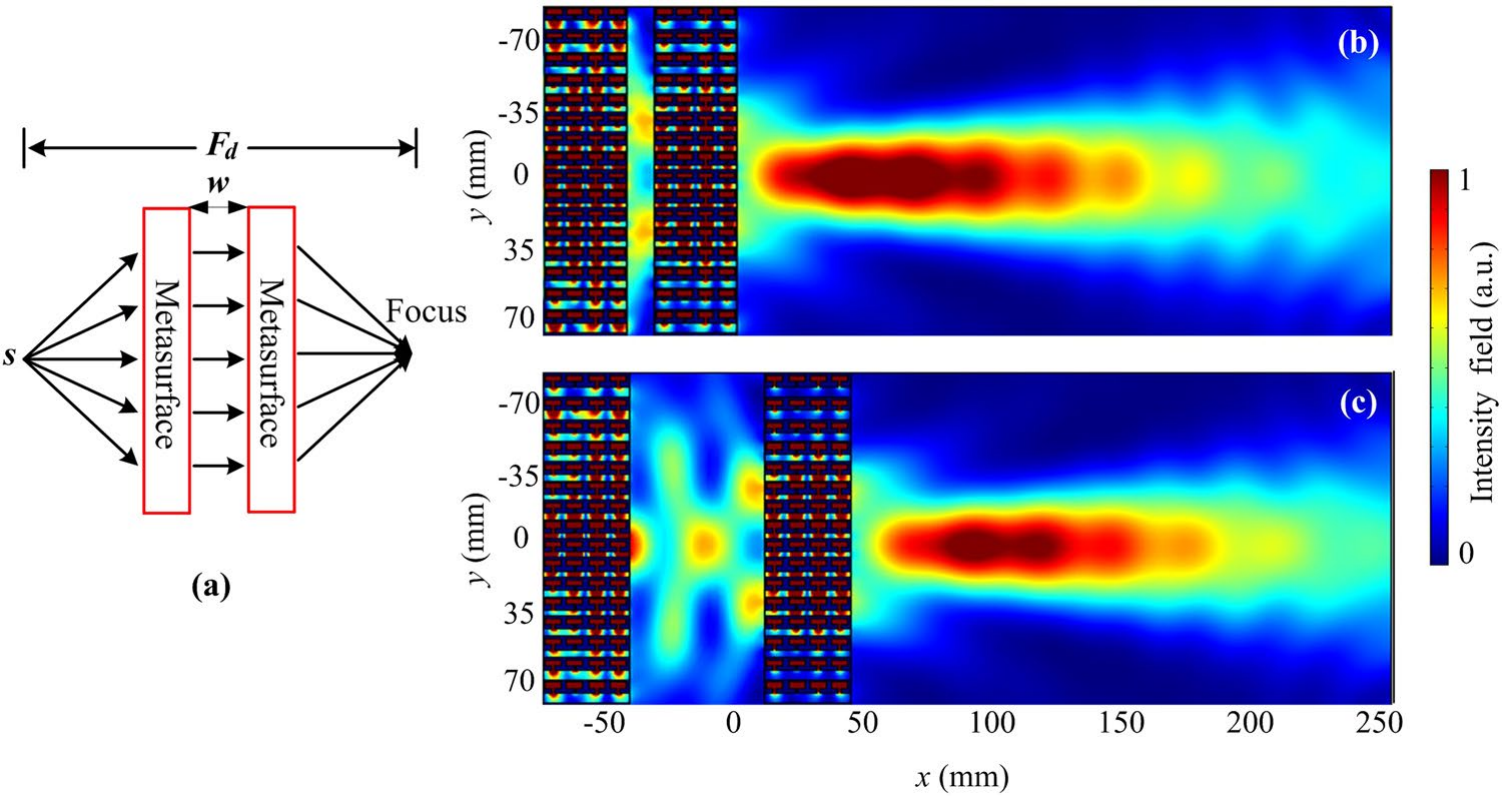
Acoustic metasurface for effective acoustic negative refraction. (**a**) The concept schematic of the conversion process of generating effective acoustic negative refraction. (**b**) and (**c**) represent the simulated pressure intensity field distributions of the composite metasurfaces with the distances *w* = 10 mm and 50 mm, respectively.

Based on the above discussions, our gradient velocity metasurfaces show the ability of controlling wavefront conversions: anomalous refraction, non-diffracting Bessel beam, and sub-wavelength flat focusing. Furthermore, as our gradient metasurfaces have high efficiency transmission and flat geometry, the proposed metasurfaces with acoustic focusing characteristic can be cascaded to achieve effective acoustic negative refraction. Figure 7a presents the concept schematic of the composite metasurface for effective acoustic negative refraction, which is constructed by directly cascading two metasurfaces of sub-wavelength flat lens demonstrated above. The corresponding simulated pressure intensity field distribution at the frequency 6970 Hz is illustrated in Fig. 7b, it is observed that a cylindrical wave emitted from a point source is refocused after propagating through the composite metasurface, which confirms the effect of effective acoustic negative refraction. Most of the previous acoustic metasurfaces manipulate acoustic wavefronts in a static pattern, which leads to restrict the practical application. Here, since this effective acoustic negative refraction can be generated by cascading two parallel metasurfaces, through adjusting the distance *w* of the two cascaded metasurfaces, the focal depth *F*_*d*_ can be modulated in a wide range. Figures 7b and 7c illustrate the simulated pressure intensity field distributions of the composite metasurfaces with the different distances *w* = 10 mm and 50 mm, respectively. When *w* = 10 mm, the focal depth is 98 mm. Then we calculate the focal depths at different distances between two cascaded metasurfaces, it is found that as the distance *w* increases linearly from 10 mm to 300 mm, the focal depth also increases from 98 mm to 340 mm, which means the effective acoustic negative refractive is flexible and tunable.

## Discussion

In summary, we have designed the gradient velocity acoustic metasurfaces that can produce arbitrary complex modulations of the wavefronts. On the basis of Helmholtz resonator unit cell, the metasurfaces are shown to efficiently redirect the refracted waves as described by the generalized Snell’s law. By carefully selecting the slit width of unit cell, the assembled metasurface can exhibit effective acoustic velocity in a discrete distribution and obtain matched impedance to improve transmission efficiency. As particular examples, anomalous refraction, non-diffracting Bessel beam, sub-wavelength flat focusing, and effective acoustic negative refraction are demonstrated to confirm the excellent wavefront manipulations of the gradient metasurfaces. The designed metasurfaces possess superior properties, such as high efficiency transmission, better acoustic impedance matching, convenient modulation and simple structure. This may provide a new design methodology for efficient modifications of sound radiation pattern and acoustic wave engineering.

## Method

Throughout the paper, the numerical simulations are conducted with commercial software COMSOL Multiphysics 4.4. The design of metasurface is based on the theoretical analysis. The materials applied in the numerical simulations are air and metal (sound hard boundaries). For Figs. 3, 4 and 5, the plane wave radiation boundaries are imposed with an incident wave on the incidence boundaries. For Figs. 6 and 7, the cylindrical waves are excited by a point source and the cylindrical radiation boundary conditions are employed on the incident boundaries. The remaining boundaries of the calculating area are set to the radiation boundary conditions according to the wave shapes.
